# Estimating anatomical trajectories with Bayesian mixed-effects modeling

**DOI:** 10.1016/j.neuroimage.2015.06.094

**Published:** 2015-11-01

**Authors:** G. Ziegler, W.D. Penny, G.R. Ridgway, S. Ourselin, K.J. Friston, for the Alzheimer's Disease Neuroimaging Initiative

**Affiliations:** a Wellcome Trust Centre for Neuroimaging, Institute of Neurology, University College London, UK; b Dementia Research Centre, Institute of Neurology, University College London, UK; c FMRIB, Nuffield Dept. of Clinical Neurosciences, University of Oxford, UK; d Translational Imaging Group, Centre for Medical Image Computing, University College London, UK

**Keywords:** Brain morphology, Lifespan brain aging, Dementia, Longitudinal analysis, Multi-level models, Bayesian inference

## Abstract

We introduce a mass-univariate framework for the analysis of whole-brain structural trajectories using longitudinal Voxel-Based Morphometry data and Bayesian inference. Our approach to developmental and aging longitudinal studies characterizes heterogeneous structural growth/decline between and within groups. In particular, we propose a probabilistic generative model that parameterizes individual and ensemble average changes in brain structure using linear mixed-effects models of age and subject-specific covariates. Model inversion uses Expectation Maximization (EM), while voxelwise (empirical) priors on the size of individual differences are estimated from the data. Bayesian inference on individual and group trajectories is realized using Posterior Probability Maps (PPM). In addition to parameter inference, the framework affords comparisons of models with varying combinations of model order for fixed and random effects using model evidence. We validate the model in simulations and real MRI data from the Alzheimer's Disease Neuroimaging Initiative (ADNI) project. We further demonstrate how subject specific characteristics contribute to individual differences in longitudinal volume changes in healthy subjects, Mild Cognitive Impairment (MCI), and Alzheimer's Disease (AD).

## Highlights

• We introduce a framework for structural trajectories using Longitudinal MRI.
• Bayesian inference on trajectories is realized using Posterior Probability Maps (PPM).
• We validate the model in simulations and real MRI data from the ADNI project.

## Introduction

Magnetic Resonance Imaging (MRI) and computational morphometry have become important tools for *in-vivo* analysis of changes in healthy and pathological brain development and aging (Frisoni et al., 2010; Fjell and Walhovd, 2010). One of the most exciting research questions is the nature of variability in aging brain structure (Raz et al., 2005, 2010; Raz and Rodrigue, 2006) and function (Pudas et al., 2013; Grady, 2012) observed across individuals. Most aging studies apply cross-sectional designs, providing estimates of population average, age-related, differences via pooling within cohorts (Ziegler et al., 2012a). However, exploring the large heterogeneity of true within-subject brain changes necessarily requires repeated measures and longitudinal designs (Raz and Lindenberger, 2011).

Longitudinal assessments offer significant advantages over cross-sectional studies (for an introduction see e.g. Fitzmaurice et al., 2008). A longitudinal study is more powerful for a fixed number of subjects. It permits separation of within- and between-subject variability, and helps to ameliorate confounds. Another important advantage is that in addition to providing estimates of population average brain changes it enables a characterization of systematic differences in longitudinal trajectories among individuals. This allows researchers to identify adverse as well as protective factors that may influence healthy and pathological changes in brain anatomy and function over time (see e.g. Taki et al., 2013; Thambisetty et al., 2012; Smith et al., 2010; Debette et al., 2011; den Heijer et al., 2012). Moreover, individual subjects' trajectories are promising biomarkers for early stage diagnosis (Chetelat and Baron, 2003), tracking of disease progression (Fonteijn et al., 2012; Jedynak et al., 2012; Sabuncu et al., 2014; Donohue et al., 2014; Young et al., 2014) and monitoring of potential treatments (Douaud et al., 2013).

Crucially, longitudinal MR-based morphometry is prone to artifacts due to scanner inhomogeneities, registration inconsistency, and subtle scanner-positioning or hydration-related deformations of the brains (Schnaudigel et al., 2010; Littmann et al., 2006; Kempton et al., 2009). Sophisticated within-subject registration pipelines have been introduced recently to parameterize structural changes in an unbiased fashion (Ashburner and Ridgway, 2013; Leung et al., 2012; Lorenzi and Pennec, 2013; Holland et al., 2011; Reuter et al., 2010, 2012).

An essential difference between longitudinal and cross-sectional analysis lies in the modeling assumptions about each individual. With a single observation per subject one has to assume the process of interest is identical across subjects (using fixed-effects assumptions). In contrast, longitudinal designs allow one to parameterize individual variations in the process by including random effects (or random coefficients). Modeling repeated measurements of behavior is well established in psychology and psychometry (for review see McArdle, 2009). In the last decade, there has been a growing interest in applications of mixed-effects models in the context of neuroimaging of development (Shaw et al., 2006, 2008; Raznahan et al., 2011a,b, 2014; Schumann et al., 2010) and aging neuroscience (Lerch et al., 2005; Lau et al., 2008; Carmichael et al., 2010). Additional articles focus specifically on methods for analysis of longitudinal MRI (Resnick et al., 2000; Chan et al., 2003; Frost et al., 2004; Bernal-Rusiel et al., 2012) and voxel-wise or vertex-wise longitudinal modeling (Guillaume et al., 2014; Li et al., 2013; Skup et al., 2012; Chen et al., 2013; Bernal-Rusiel et al., 2013).

Bayesian inference has been successfully applied to functional brain scans in multiple domains, ranging from general linear models, group analysis, spatial models, analysis of connectivity, to model comparisons (for extensive review see Woolrich, 2012). Bayesian inference typically exploits hierarchical observation models that take into account different levels of observations (e.g. scans and subjects), allows for the inclusion of biologically informed prior-beliefs about parameters, and affords comparisons among competing (nested or non-nested) models. Bayesian treatment of whole-brain neuroimaging data might also increase the sensitivity by finessing the problem of multiple comparison (Friston and Penny, 2003; Schwartzman et al., 2009). In contrast to classical inference, it also enables the assessment of evidence in favor of the null hypothesis; i.e., no aging-related change or preservation of structural integrity. These issues speak to a Bayesian framework for modeling structural change trajectories. However, there are currently only a few existing studies that consider longitudinal structural MRI (Schmid et al., 2009; Chen et al., 2012).

Here, we propose a generic modeling framework for longitudinal morphometric brain changes in development and aging studies. After diffeomorphic registration on the within-subject (Ashburner and Ridgway, 2013) and between-subject (Ashburner and Friston, 2011) level, we build a generative linear mixed-effects model of repeated observations. The model inversion flexibly accommodates unbalanced and sparse designs with potentially different numbers of follow up scans per subject. Using Expectation Maximization (EM) we obtain voxelwise individual and group level change parameters and compute Posterior Probability Maps (PPM) (Friston and Penny, 2003) for inference about regionally specific effects. In other words, we focus on making regionally specific inferences about longitudinal changes in anatomy, that properly account for both within and between subject variability in neurodevelopmental trajectories.

We validate the model using simulated data and a large MRI sample from the ADNI cohort. We then demonstrate a parametric analysis of subject specific covariates and explore the model space to optimize explanations of individual trajectory differences.

## Methods

In this section, we introduce a generative model of local structural trajectories using random and fixed effects; i.e., a mixed effect, hierarchical or multilevel model. We describe the Bayesian formulation, the implicit (empirical) prior covariance components and their estimation using EM. We extend this framework to modeling of trajectories over multiple groups and review the use of PPMs for inference on model parameters. We conclude this section with a treatment of Bayesian model selection of ensemble trajectory models.

### A generative model of local structural trajectories

The model for age-related changes of local brain structure (per voxel or region) is based upon the following generative model, which comprises a likelihood and prior. The model is an application of the Bayesian linear hierarchical observation framework introduced by Friston et al. (2002a) (for application in the context of fMRI see also Friston et al., 2002b).

We here consider the special case of a two level model, one for individual structural trajectories and a second level for an ensemble of trajectories, denoted by *ε*. The first level likelihood model is based on the assumption that the trajectory of underlying volumetric changes is sampled from subject-specific functions of age or time

(1) $$y_{ij}=g\left(t_{ij},\theta_{i}^{\left(1\right)}\right)+\epsilon_{ij}^{\left(1\right)}$$

where the measurement *y_ij_* is the *j*-th of *m_i_* observations (e.g. of gray matter density at a single voxel) obtained from the *i*-th of *N* subjects at age *t_ij_*, and ϵ_*ij*_^(1)^ denotes an i.i.d. Gaussian measurement error with variance *σ*^2^. In what follows we use time centered *t_ij_* in order to develop trajectories around the reference age, i.e. *t_r_*, which typically is chosen as the mean age of the sample. Individual differences of trajectories are thus encoded by subject-specific change parameters ***θ***_*i*_^(1)^ resulting in an ensemble of age-related trajectories *ε* = {*g*(*t*, ***θ***_*i*_^(1)^)}_*i* = 1_^*N*^ for a sample of individuals. In particular, we parameterize the function describing the trajectory using a *D* degree polynomial expansion of age

(2) $$g\left(t,\theta_{i}^{\left(1\right)}\right)=\sum_{d=1}^{D+1}\;\theta_{di}^{\left(1\right)}t^{d-1}$$

with coefficients ***θ***_*i*_^(1)^ = [*θ*_1,*i*_^(1)^, …, *θ*_*D* + 1,*i*_^(1)^]^*T*^. For example, for *D* = 2 we have 3 coefficients per subject, encoding the intercept, slope and quadratic terms. We can easily write these linear models using compact matrix notation with individual design matrices and change parameters as **g**_*i*_ = **X**_*i*_^(1)^***θ***_*i*_^(1)^. Then, the model for all subjects follows

(3) $$\left[\begin{array}{c} y_{1}\\ y_{2}\\ \vdots \\ y_{N} \end{array}\right]=\left[\begin{array}{cccc} X_{1}^{\left(1\right)} & & & \\ & X_{2}^{\left(1\right)} & & \\ & & \ddots & \\ & & & X_{N}^{\left(1\right)} \end{array}\right]\;\left[\begin{array}{c} \theta_{1}^{\left(1\right)}\\ \theta_{2}^{\left(1\right)}\\ \vdots \\ \theta_{N}^{\left(1\right)} \end{array}\right]+\epsilon^{\left(1\right)}$$

(4) $$y=X^{\left(1\right)}\theta^{\left(1\right)}+\epsilon^{\left(1\right)}$$

with subject *i*'s observations $y_{i}={\left[y_{i1},y_{i2},\ldots ,y_{im_{i}}\right]}^{T}$, *M* = ∑ *m*_*i*_ concatenated observations *y*, first level design matrix **X**^(1)^, concatenated change parameters ***θ***^(1)^, and first level Gaussian errors **ϵ**^(1)^. Vectorizing observations *y_ij_* in ‘person-scan’ format, i.e. the successive scans are grouped by subjects (all from subject 1, all from subject 2, etc.), is a natural way to arrange longitudinal data with missing scans and varying number of follow ups. This additionally simplifies the structure of the first level design matrix, which then takes a block-diagonal form. Note, that this first level model explicitly accommodates unbalanced designs, i.e. **X**_*i*_^(1)^ ≠ **X**_*j*_^(1)^, with varying ages and numbers of scans per subject.

The sample change parameters of the trajectory functions are determined by (primarily non-age-dependent) subject specific effects. Note that these second level regressors can be chosen to model covariates of interest, e.g. IQ scores, genetic markers, or symptom severity, as well as purely confounding variables, e.g. global brain parameters. These measures are summarized in a centered *N* × *R* between-subject covariates matrix **Z** with entries *z_ir_*. For example, in the results section below, we use a genetic risk score as a covariate of interest and test to see how this predicts first level parameters. Now, we adopt the following linear second level model

(5) $$\left[\begin{array}{c} \theta_{1}^{\left(1\right)}\\ \theta_{2}^{\left(1\right)}\\ \vdots \\ \theta_{N}^{\left(1\right)} \end{array}\right]=\left[\begin{array}{cccc} I & z_{11}I & & z_{1R}I\\ I & z_{21}I & & z_{2R}I\\ \vdots & & \ddots & \vdots \\ I & z_{N1}I & & z_{NR}I \end{array}\right]\;\left[\begin{array}{c} \theta_{1}^{\left(2\right)}\\ \theta_{2}^{\left(2\right)}\\ \vdots \\ \theta_{R+1}^{\left(2\right)} \end{array}\right]+\epsilon^{\left(2\right)}$$

(6) $$\theta^{\left(1\right)}=X^{\left(2\right)}\theta^{\left(2\right)}+\epsilon^{\left(2\right)}$$

with *D* + 1 dimensional identity matrix **I**, second level design matrix **X**^(2)^, concatenated parameters ***θ***^(2)^, and zero mean multivariate Gaussian errors **ϵ**^(2)^ respectively (for distributions details see also the section on Covariance component specification). Note, that we can further simplify the structure of the design matrix by writing it as a Kronecker product [[1_*N*_**Z**] ⊗ **I**_*D* + 1_] using N dimensional column vector of ones 1*_N_*. Although one could choose a separate set of covariates for each trajectory parameter, we here consider the common exploratory situation where one is interested in potential effects of a small set of covariates on all trajectory properties, i.e. intercept, slope, etc.

Due to the particular choice of a column of ones in the second level design, it follows that ***θ***_1_^(2)^ parameterizes the sample average change in terms of a mean trajectory, which is the expectation for every subject's trajectory parameters after accounting for covariate effects. The remaining second level parameters ***θ***_2_^(2)^, …, ***θ***_*R* + 1_^(2)^ become the coefficients of each covariate's contribution to individual trajectory differences.

### Combining fixed and random effects

The above model with degree zero might be referred to as the random intercept model without slope. Using this model in the context of longitudinal MRI assumes variability of structure across subjects but no changes over time. If we chose model degree one, the model now includes a random slope parameter for every subject. One might argue that the first (or higher) degree(s) can also enter as fixed (as opposed to random effects); e.g., assuming the same rate of change (or quadratic effect) for all subjects. The above framework naturally extends to modeling these additional fixed effects of degree *d* by appending column vectors **x**_*f*_^*d*^ with entries $t_{im_{i}}^{d}$ to the first level design matrix $\left[X^{\left(1\right)},x_{f}^{D+1},\ldots ,x_{f}^{D_{f}}\right]$. In this case we need to extend first level parameters accordingly, i.e. ***θ***^(1)^ = [***θ***_1_^(1)^, …, ***θ***_*N*_^(1)^, ***θ***_*f*_^(1)^]. In presence of these fixed effects the second level design follows as

(7) $$\left[\begin{array}{l} \theta^{\left(1\right)}\\ \theta_{f}^{\left(1\right)} \end{array}\right]=\left[\begin{array}{cc} X^{\left(1\right)} & 0\\ 0 & I_{D_{f}-D} \end{array}\right]\left[\begin{array}{l} \theta^{\left(2\right)}\\ \theta_{f}^{\left(2\right)} \end{array}\right]+\left[\begin{array}{l} \epsilon^{\left(2\right)}\\ \epsilon_{f}^{\left(2\right)} \end{array}\right]\text{.}$$

If we now constrain the second level errors for fixed effects parameters to be zero, we can perform second level group inference for random and fixed effects parameters in a similar way (as will be shown in the Bayesian perspective section). In what follows we use *D* to denote the degree of random effects and *D_f_* for the degree of fixed effects. Please note that entering fixed effects, in addition to random effects of the same degree, would result in redundant parameters for the average trajectory. Thus, one might prefer only using additional fixed effects with higher degrees *D_f_* > *D*. This parametrization of fixed and random effects is motivated by our hierarchical formulation of the model and might slightly differ from standard mixed-effects textbooks.

### Covariance component specification

In order to estimate the above model, we need to fully specify all covariance constraints for first and second level errors, further denoted with **C**_ϵ_^(1)^ and **C**_ϵ_^(2)^ respectively. Given an unknown covariance structure **C** we use a small set of covariance basis functions **Q***_k_* and estimate the corresponding coefficients or hyperparameters *λ_k_*

(8) $$C\left(\lambda \right)=\sum_{k}\lambda_{k}Q_{k}\text{.}$$

More generally, this can be motivated by a first-order Taylor expansion of the covariances with respect to their hyperparameters (for details see e.g. Friston et al., 2002a). This idea will be now outlined for all covariance components of the above model. We begin with a single group design, and extend this to modeling of multiple groups in later sections. It is important to note that the covariance could be specified being linear in the hyperparameters (as seen in Eq. (8)); however, this does not preclude negative definite covariances (Harville, 1977). In contrast to optimizing linear coefficients *λ*, in what follows, we optimize log-covariance parameters, ie. *e^λ^*. This forces the hyperparameters to be positive and at the same time increases the stability of the subsequent optimization scheme.

In particular, as mentioned above, we specify the first level error covariance using an isotropic noise model

(9) $$C_{\epsilon }^{\left(1\right)}=e^{\log\left(\sigma^{2}\right)}I_{M}$$

with **I***_M_* denoting an identity matrix and noise variance *σ*^2^. This models unstructured errors of measurement, e.g. due to MRI noise and random errors or minor inaccuracies during preprocessing. Furthermore we recall that every subject is fully described by its parameter vector ***θ***_*i*_^(1)^. Considering the population, however, there is unknown variability of individual parameters across subjects, which is either explicitly modeled by covariates (or group structures) in design matrix **X**^(2)^ or captured by the second level error covariance **C**_ϵ_^(2)^. The unexplained individual differences might differentially affect all trajectory coefficients and thus (at least) one further hyperparameter for each of the trajectory parameters is required. We therefore use *λ*_1_, *λ*_2_, etc. to describe unexplained individual differences of intercept, slope etc. For that purpose we use **R***_i_* to denote the covariance matrix of residual parameter vectors *Cov*(**ϵ**_*i*_) and we suppose

(10) $$R_{i}=\left[\begin{array}{lll} e^{\lambda_{1}} & & \\ & \ddots & \\ & & e^{\lambda_{D+1}} \end{array}\right]\text{.}$$

Typically, having only very sparse observations in longitudinal MRI designs prevents us from estimating **R***_i_* on the individual level. For reasons of identifiability in a wide range of designs, we therefore assume the same residual covariance across all subjects, i.e. **R_i_** = **R**. The full second level error covariance can be therefore specified as follows

(11) $$C_{\epsilon }^{\left(2\right)}=\left[\begin{array}{lll} R & & \\ & \ddots & \\ & & R \end{array}\right]=I_{N}⊗R=\sum_{d=1}^{D+1}e^{\lambda_{d}}Q_{d}$$

where covariance basis functions **Q***_d_* can be efficiently implemented exploiting the Kronecker product. Taken together [*σ*^2^, *λ*_1_, …, *λ*_*D* + 1_] fully parameterize the covariance components of the model in its simples form; resulting, e.g. in three voxelwise hyperparameters for single ensembles of linear trajectories. Please note that the above framework nicely extends to more complex models, e.g. with first level covariates and correlated residuals at the second level.

Finally, we finesse the covariance components to account for any fixed effects as discussed in the section on Combining fixed and random effects. This means we consider the case when the degree of fixed effects exceeds the degree of random effects and we apply extended design matrices and parameters (Eq. (7)). In order to perform similar inference for second level fixed effects parameters like group average parameters of random effects we enforce identity of first and second level fixed effects parameters, i.e. ***θ***_*f*_^(1)^ = ***θ***_*f*_^(2)^. This can be easily implemented by choosing a hyperparameter of second level fixed effects errors with a very small variance, i.e. $\epsilon_{f}^{\left(2\right)}\sim N\left(0,\sigma_{f}^{2}I_{D_{f}-D}\right)$ with e.g. *σ*_*f*_^2^ = *e*^− 32^.

### Bayesian perspective

We now explore the Bayesian perspective on the above model for an ensemble of trajectories (defined by Eqs. (4) and (6)). A key aspect of this formulation is that we can consider the second level to furnish an empirical prior for the first level parameters, as follows

(12) $$P\left(y\left|\theta^{\left(1\right)}\right)\right)=N\left(y;X^{\left(1\right)}\theta^{\left(1\right)},C_{\epsilon }^{\left(1\right)}\right)$$

(13) $$P\left(\theta^{\left(1\right)}\left|\theta^{\left(2\right)}\right)\right)=N\left(\theta^{\left(1\right)};X^{\left(2\right)}\theta^{\left(2\right)},C_{\epsilon }^{\left(2\right)}\right)$$

with error covariances **C**_ϵ_^(*k*)^, *k* = 1, 2. The first level error covariance corresponds to measurement noise.

Finally, we assume second level priors on the ensemble change parameters. At the end of this section we will briefly discuss promising choices of priors which might be relevant for potential applications:

(14) $$P\left(\theta^{\left(2\right)}\right)=N\left(\theta^{\left(2\right)};\eta_{\theta }^{\left(2\right)},C_{\theta }^{\left(2\right)}\right)\text{.}$$

The hierarchical structure of the trajectory model implies that the joint probability factorizes as

(15) $$P\left(y,\theta^{\left(1\right)},\theta^{\left(2\right)}\right)=P\left(y\left|\theta^{\left(1\right)}\right)\right)P\left(\theta^{\left(1\right)}\left|\theta^{\left(2\right)}\right)\right)P\left(\theta^{\left(2\right)}\right)$$

rendering the data conditionally independent of the second level parameters given the first level parameters (Bishop, 2006).

In this framework, hierarchical model inversion corresponds to estimating covariance components **C**_ϵ_^(1)^, **C**_ϵ_^(2)^ and **C**_*θ*_^(2)^ respectively. For this purpose, the model can be further rearranged in a non-hierarchical form (see also Friston et al., 2002a)

(16) $$y=\left[\begin{array}{cc} X^{\left(1\right)} & X^{\left(1\right)}X^{\left(2\right)} \end{array}\right]\left[\begin{array}{l} \epsilon^{\left(2\right)}\\ \theta^{\left(2\right)} \end{array}\right]+\epsilon^{\left(1\right)}\text{.}$$

Exploiting the Bayesian perspective, we treat the second level errors as additional model parameters, which will be estimated in subsequent steps.

To ensure all covariance components are evaluated simultaneously, we further augment the model by adding rows that correspond to the prior expectation *E*[**ϵ**^(2)^] = 0 and *E*[***θ***^(2)^] = ***η***_*θ*_^(2)^ respectively

(17) $$\left[\begin{array}{c} y\\ 0\\ \eta_{\theta }^{\left(2\right)} \end{array}\right]=\left[\begin{array}{cc} X^{\left(1\right)} & X^{\left(1\right)}X^{\left(2\right)}\\ I & 0\\ 0 & I \end{array}\right]\left[\begin{array}{c} \epsilon^{\left(2\right)}\\ \theta^{\left(2\right)} \end{array}\right]+\left[\begin{array}{c} \epsilon^{\left(1\right)}\\ -\epsilon^{\left(2\right)}\\ \eta_{\theta }^{\left(2\right)}-\theta^{\left(2\right)} \end{array}\right]$$

(18) $$\bar{y}=\bar{X}\theta +\bar{\epsilon }$$

with augmented data $\bar{y}$, augmented design $\bar{X}$ and parameters ***θ*** and augmented errors $\bar{\epsilon }$. Note that in contrast to the models considered above the augmented error contains all covariance components of the two-level model. One further benefit of augmentation is that it allows formulating the Gaussian likelihood and prior of the ensemble trajectories in a pleasingly compact form

(19) $$p\left(\bar{y}\left|\theta \right)\right)=N\left(\bar{y};\bar{X}\theta ,C_{\epsilon }\right)$$

(20) $$p\left(\theta \right)=N\left(\theta ;\eta_{\theta },C_{\theta }\right)$$

with expectation and covariance components

(21) $$\eta_{\theta }=\left[\begin{array}{c} 0\\ \eta_{\theta }^{\left(2\right)} \end{array}\right],C_{\epsilon }=\left[\begin{array}{cc} C_{\epsilon }^{\left(1\right)} & 0\\ 0 & C_{\theta } \end{array}\right],C_{\theta }=\left[\begin{array}{cc} C_{\epsilon }^{\left(2\right)} & 0\\ 0 & C_{\theta }^{\left(2\right)} \end{array}\right]\text{.}$$

Longitudinal MRI studies of healthy and pathological development rest on inferences about first or second level parameters of the above model. The posterior density over parameters, given a particular sample of observations, is also Gaussian and can be written using the compact Gauss–Markov form

(22) $$P\left(\theta \left|y\right)\right)=N\left(\theta ;\eta_{\theta |y},C_{\theta |y}\right)\mathrm{with}$$

(23) $$C_{\theta |y}={\left({\bar{X}}^{T}C_{\epsilon }^{-1}\bar{X}\right)}^{-1}\mathrm{and}$$

(24) $$\eta_{\theta |y}=C_{\theta |y}\left({\bar{X}}^{T}C_{\epsilon }^{-1}y\right)\text{.}$$

The ensuing model inversion can be performed in a fully Bayesian way, i.e. using an informative prior on top level parameters; i.e., with given ***η***_*θ*_^(2)^ and **C**_ϵ_^(2)^. These prior distributions can be specified based on expectations from the literature or as suggested in Friston and Penny (2003) one might apply empirically derived prior distributions using the data at hand, e.g. obtained from a pooled covariance estimate. Moreover, if one does not have explicit prior assumptions about the local patterns of change, one can treat these parameters as unknown, thus using uninformative priors.

**Fig. 1:**
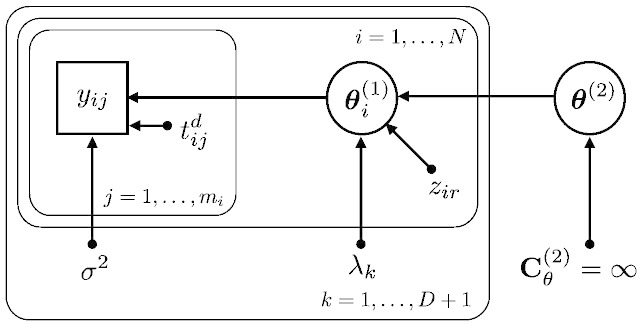
Illustration of the trajectory model using a directed graphical model emphasizing the Bayesian perspective. Rectangles are used for observed variables, e.g. *y_ij_* is the *j*-th observation of the *i*-th subject. Ellipsoids are used for latent (or hidden) stochastic variables, e.g. ***θ***_*i*_^(1)^ refers to intercept, slope, etc. of the *i*-th subject. ***θ***^(2)^ denotes all second level parameters, e.g. all groups' average intercept, slope, and covariate effects. All other parameters with arrows denote deterministic variables, e.g. *z_ir_* is the *r*-th covariate for the *i*-th subject or the timepoint *t_ij_* of the *j*-th observation of the *i*-th subject. For the top level parameters, we apply flat priors denoted by an infinite prior variance parameter. We have introduced plates that compactly represent multiple variables (and arrows), for which only a single example is shown explicitly.

In this particular study we apply uninformative or flat priors with **C**_*θ*_^(2)^ = ∞ (or equivalently (**C**_*θ*_^(2)^)^− 1^ = 0), with the prior expectation ***η***_*θ*_^(2)^ set to zero. In order to obtain the posterior over all trajectory parameters, we estimate the covariance components using an EM scheme. As described above, the top level prior covariance is unknown, realized by setting it to an arbitrarily high value, in particular we choose **C**_*θ*_^(2)^ = *e*^32^**I**. A simple illustration of the applied model is shown in Fig. 1.

### Model estimation using Expectation Maximization

As proposed by Friston et al. (2002a) we adapt an EM algorithm (Dempster et al., 1977) to obtain all covariance components and the posterior of the change parameters. EM iteratively refines a lower bound *F* on the log-likelihood of the data given the hyperparameters, i.e. *ln p*(*y*|***λ***) ≥ *F*(*q*(***θ***), ***λ***), where *q*(***θ***) is any distribution of the change parameters. Using iterative alternation between E and M steps (see later), one performs a coordinate ascent on F, and thus implicitly increases the log-likelihood.

#### E-Step

Under the above Gaussian assumptions, each E-step maximizes *F*(*q*(***θ***), ***λ***) with respect to the distribution *q*(*θ*). Here, this simply corresponds to obtaining sufficient statistics for the posterior of the parameters, i.e. *F* is maximized by *q*(***θ***) = *p*(***θ***|*y*, ***λ***). Using the covariance parametrization of the augmented model and Eqs. (23) and (24) the posterior is given by

(25) $$\eta_{\theta \left|y\right)}=C_{\theta \left|y\right)}{\bar{X}}^{T}C_{\varepsilon }^{-1}y\;\mathrm{with}$$

(26) $$C_{\theta \left|y\right)}={\left({\bar{X}}^{T}C_{\epsilon }^{-1}\bar{X}\right)}^{-1}\mathrm{and}$$

(27) $$C_{\epsilon }=C_{\theta }+\sum_{k}e^{\lambda_{k}}Q_{k}\text{.}$$

#### M-Step

Here, we optimize *F*(*q*(***θ***), ***λ***) with respect to the covariance hyperparameters, in a maximum likelihood sense, using the posterior distribution obtained during the preceding E-step. In particular, *F* during the M-step is given by

(28) $$F=\frac{1}{2}\ln\left|C_{\epsilon }^{-1}\right|-\frac{1}{2}r^{T}C_{\epsilon }^{-1}r-\frac{1}{2}\mathrm{tr}\left(C_{\theta |y}\bar{X}C_{\epsilon }^{-1}\bar{X}\right)+\frac{1}{2}\ln\left|C_{\theta \left|y\right)}\right|+\mathrm{const}$$

with residuals $r=\bar{y}-\bar{X}\eta_{\theta \left|y\right)}$ (for an exact derivation see Friston et al., 2002a). The first term decreases *F* with a larger number and size of hyperparameters, while the second term increases *F* with smaller precision weighted residuals corresponding to a better model fit.

Note also, that during the M-step the posterior covariance **C**_*θ*|*y*_ is a fixed result from the preceding E-step, while **C**_ϵ_ = **C**_ϵ_(***λ***) depends on the hyperparameters and will be optimized. Thus in general the third term of *F* is not the trace of an identity matrix. The last term, which stems from the entropy of the distribution over change parameters *q*(*θ*), can be neglected, because it does not depend on the hyperparameters.

To update the hyper parameters we adopt a Fisher scoring algorithm, using the first derivative (or gradient) *g* and the expected second partial derivatives (or Fisher's Information matrix) **H**:

(29) $$\lambda =\lambda +H^{-1}g\;\mathrm{with}$$

(30) $$g_{k}=\frac{\partial F}{\partial \lambda_{k}}=-\frac{1}{2}e^{\lambda_{k}}\left(tr\left(PQ_{k}\right)-{\bar{y}}^{T}P^{T}Q_{k}P\bar{y}\right)\text{,}$$

(31) $$H_{\mathrm{kl}}=E\left[\frac{\partial^{2}F}{\partial \lambda_{k}\partial \lambda_{l}}\right]=\frac{1}{2}e^{\lambda_{k}+\lambda_{l}}tr\left(PQ_{k}PQ_{l}\right),\;\mathrm{and}$$

(32) $$P=C_{\epsilon }^{-1}-C_{\epsilon }^{-1}\bar{X}C_{\theta \left|y\right)}{\bar{X}}^{T}C_{\epsilon }^{-1}\text{.}$$

The updated hyperparameters re-enter into the estimation of the posterior in the next E-step.^2^ Finally, after appropriate initialization of the hyperparameters ***λ***, the full algorithm alternates between the E- and M-steps until convergence.

### Multiple groups

Longitudinal studies of development and aging often aim at inference about differences among average population trajectories. Typically, this involves comparing change rates (or slope differences) in healthy vs. pathological development, specific treatment conditions, or groups following specific lifestyle patterns. Although the ongoing structural change is well characterized by the slope parameters, the current framework also supports inference about other aspects of trajectory shape; e.g., intercepts or higher order non-linearities.

We therefore generalize the above model to situations where one observes *M* scans of *N* = *N*_1_ + *N*_2_ + … + *N*_*G*_ subjects, who are individuals from *G* different populations with (mainly non-age dependent) subject-specific covariates **Z**_1_, …, **Z**_*G*_. For example, we might consider three groups of subjects in the ADNI dataset, including controls, MCI and AD, with Mini Mental State Examination scores as covariates. The subsamples are further used to estimate independent ensembles of trajectories *ε*_1_, …, *ε*_*G*_ using the same trajectory parametrization (Eq. (4)), e.g. quadratic curves. The assumptions about the group structure of trajectories can be realized by modifying the second level design matrix (in Eq. (6)) appropriately

(33) $$X^{\left(2\right)}=\left[\begin{array}{llll} 1_{N_{1}}\;Z_{1} & & & \\ & 1_{N_{2}}\;Z_{2} & & \\ & & \ddots & \\ & & & 1_{N_{G}}\;Z_{G} \end{array}\right]⊗I_{D+1}\text{.}$$

In addition to allowing for different average trajectories in different groups, the amount of individual differences within each ensemble *ε*_*g*_ might also differ across populations. This is easily achieved by adapting the model of the second level covariance components to include independent hyperparameters for each group.

We suppose the covariance structure of second level residuals to be Cov(**ϵ**_*i*_^(2)^) = **R**_*g*_ for subject *i* from group *g*. We again exploit diagonal covariance basis functions ***Q**_d_* (see the section on Covariance component specification) to parameterize the variability of all change parameters in all groups resulting in *G*(*D* + 1) hyperparameters for the second level model

(34) $$C_{\epsilon }^{\left(2\right)}=\left[\begin{array}{llll} I_{N_{1}}⊗R_{1} & & & \\ & I_{N_{2}}⊗R_{2} & & \\ & & \ddots & \\ & & & I_{N_{G}}⊗R_{G} \end{array}\right]=\sum_{d=1}^{G\left(D+1\right)}e^{\lambda_{d}}Q_{d}\text{.}$$

Note that one could include fixed effects of time or age. In many practical applications these would enter as group specific fixed effects for each group and trajectory parameter. Finally, having specified a single or multi-group trajectory model, the estimation of parameters and covariance components proceeds using EM as described above.

### Inference about group differences and analysis of individual differences of change

To facilitate practical applications to longitudinal MRI studies, we also need to consider Bayesian inference about population differences and subject specific covariate effects on individual trajectories. These effects can be characterized using the usual approach of defining contrasts for linear models as commonly used in Statistical Parametric Mapping (SPM) (Friston et al., 1995). In particular, single contrast vectors are used to specify a single hypothesis about first or second level change parameters. For example, let us suppose a design with linear trajectories (first level) and two groups and no covariates (second level). If we use contrast vector **c** = [0, 1, 0, − 1]^*T*^, then **c**^*T*^***θ***^(2)^ = 0 tests the (null) hypothesis that the rate of change (slope) in group one is equal to the slope in group two. Moreover, multiple contrast vectors can be used to specify compound hypotheses. If **c**_1_ = [1, 0, − 1, 0]^*T*^, and **c**_2_ = [0, 1, 0, − 1]^*T*^ then [**c**_1_, **c**_2_]^*T*^***θ***^(2)^ = **0** assumes both intercepts and slopes to be same across groups.

PPMs were introduced for Bayesian inference on mass-univariate general linear models used in neuroimaging (Friston and Penny, 2003). When applying PPMs, one is often interested in the probability of linear contrasts *c* = **c**^*T*^***θ***^(2)^ exceeding a certain threshold, e.g. *γ* = 0. One can additionally specify a nonzero probability threshold, typically *p_t_* = 0.95. We are now in a position to construct PPMs for Bayesian inference on arbitrary trajectory parameter contrasts by voxelwise evaluation of the posterior

(35) $$p\left(c>\gamma |y\right)=1-\phi \left(\frac{\gamma -c^{T}\eta_{\theta \left|y\right)}}{\sqrt{c^{T}C_{\theta \left|y\right)}c}}\right)>p_{t}$$

with the cumulative density function of the unit normal distribution *ϕ*. Similarly, this framework affords comparison of structural change of single individuals using the first level individual change parameters ***θ***^(1)^.

### Comparison of different trajectory models

The above framework for individual trajectory estimation requires an *a-priori* assumption about the polynomial order of random or fixed effects. Generally, comparing various trajectory models corresponds to the evaluation of competing hypotheses about trajectories in development, aging and pathology or about nonlinear changes during the lifespan. One can also use model comparison to test for differences among groups, e.g. H0: all subjects in same group vs. H1: subjects in control, MCI and AD groups. Crucially, one can use Bayesian model comparison to optimize aspects of the models about which one is uncertain such as the degree order of the polynomial above. Practically, Bayesian model comparison rests upon model evidence that is approximated by the free energy obtained from EM. This (lower bound) approximation to log model evidence is used to monitor convergence during parameter estimation of any particular model and optimize the model *per se*.

Bayesian model comparison has been suggested as a principled approach for inference about nested and non-nested models of neuroimaging data (Penny et al., 2004; Penny, 2012). Assuming the same prior probability for both model orders, different orders can compared using the difference in free energy or log evidence. This corresponds to the log Bayes factor (Kass and Raftery, 1995). Local voxelwise evaluation of this probability ratio compares model evidences of models with different degrees

(36) $$BF=\frac{P\left(y\left|D=i\right)\right)}{P\left(y\left|D=j\right)\right)}$$

or models with and without some additional fixed effects. Observing BF > 1 in the above example indicates that it is more likely that individual differences of change are better captured by order *i* compared to *j*.

Questions about model order can be addressed flexibly using nested model comparisons. Two models are nested when the smaller (e.g. linear) model is obtained by setting some parameters of the larger (e.g. quadratic) model to zero. Note however, that this comparison of model evidence naturally extends to non-nested models, e.g. comparing two models with two different sets of covariates.

### Summary of methods

In summary, we propose a hierarchical generative model to infer families of (nonlinear) trajectories reported by longitudinal changes in local brain volumes (or tissue densities). The key aspect of this model is its hierarchical structure, wherein the first (within-subject) level accommodates longitudinal effects whose trajectory depends upon group average parameters at the second (between-subject) level. Crucially, this level includes differences in subjects that may be of interest; for example, group differences or diagnosis, behavior or genetic variables (see later). Alternatively, second level effects may be considered as confounds; for example, the age of a subject (e.g., at baseline), their gender, or brain/head size (Barnes et al., 2010). By modeling nonlinear trajectories in this fashion, one can easily accommodate unbalanced designs, while exploiting the efficiency of mixed-effects inference and associated parameter estimates.

## Results

### Validation using simulated structural trajectories

In what follows, we address the face validity of the above approach using simulated data generated by the model with linear trajectories drawn from the range of design and (hyper-) parameter specifications typical of longitudinal MRI and VBM preprocessing. The simulated data were entered into EM to compare parameter estimates with the ground truth. This basically establishes that the model inversion can recover veridical parameter estimates. This validation procedure followed two steps.

**Fig. 2:**
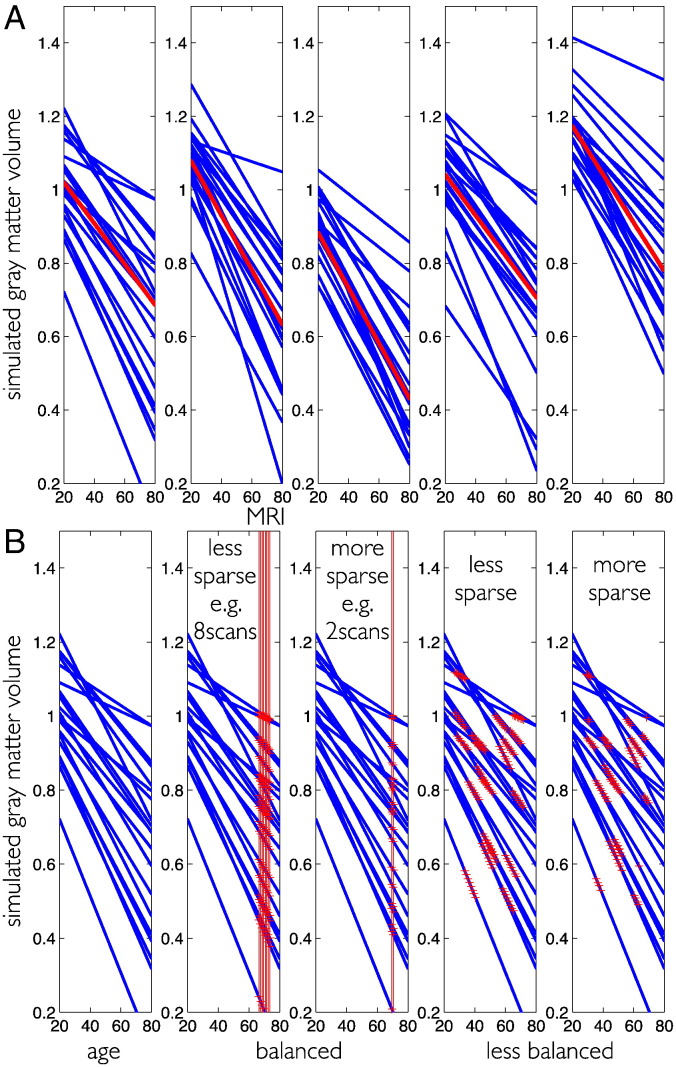
Illustration of ground truth simulation for structural trajectories. (A) 5 random realizations of an ensemble of linear trajectories are plotted over the adult lifespan. Individual trajectories are shown in blue and the average trajectory is shown in red. (B) Illustration of simulated MRI acquisition. Ages of measurement *t_ij_* are depicted by red crosses and red lines. Balanced designs (2 and 3 from left) vs. unbalanced (4 and 5 from left) and low (2nd from left) vs. high (3rd from left) sparsity of observations. Unbalanced sampling is illustrated using the age interval [20, 70] but see text for exact specification of the simulation.

Firstly, simulation of an ensemble of trajectories corresponding to a set of parameters ***θ***^(1)^ with specified average and individual trajectory differences. In the above generative model, this corresponds to the case of having only a column of ones in the second level design. Second level average change parameters were fixed to ***θ***^(2)^ = [1.2, − 5 ⋅ 10^− 3^]^*T*^, i.e. the mean intercept is 1.2 and mean slope is − 5 ⋅ 10^− 3^. No subject covariates were included in these simulations. To evaluate model performance in different contexts, the individual differences of the intercept and the slope, i.e. [*λ*_1_, *λ*_2_], were either assumed to be large [10^− 2^, 10^− 4^] or small [10^− 4^, 10^− 6^] respectively. Illustrations of simulated trajectories are shown in Fig. 2A.

Secondly, performing longitudinal MRI acquisition is equivalent to sparse temporal sampling of the unknown ground truth trajectories. The sampling process is specified by the first level design matrix. However, longitudinal MRI studies might vary substantially with respect to two main design characteristics. Designs can be more or less balanced with respect to age and differ with respect to the number of follow up measures per subject, i.e. more or less sparse. The simulation of MRI sampling and other design factors are illustrated in Fig. 2B.

**Fig. 3:**
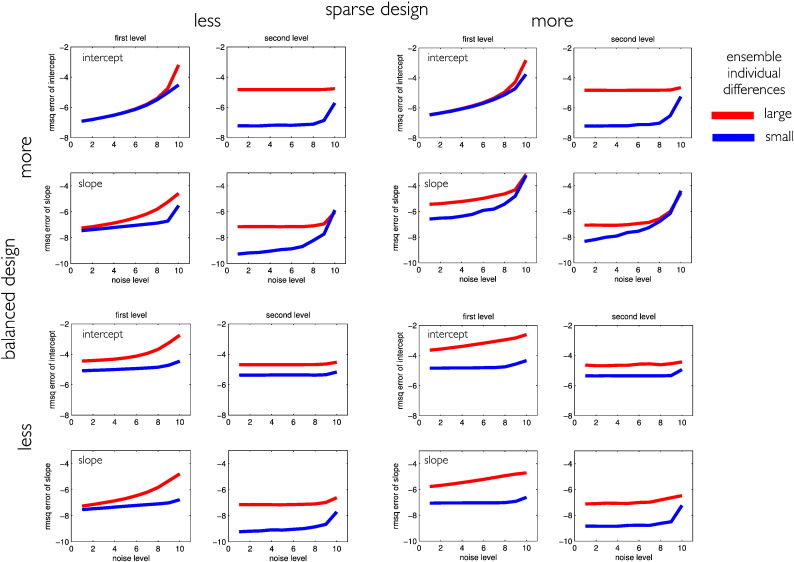
Effects of design sparsity and having more or less balanced designs for first and second level model parameter estimation accuracy. All plots show log root mean squared errors (RMSQE) comparing ground truth vs. Bayesian model parameter estimates of intercept and slope for first (individual) and second (group) level. We manipulated the noise variance to follow *σ*^2^ = 0.01/(1 + 25 × (*p* − 1)^2^), with *p* = 1, …, 10 indicating the noise level. Red vs. blue lines indicate errors for large vs. small individual differences as a function of the first level noise parameter. Stronger noise mainly increases first level model errors. The log RMSQE is depicted for different designs with independent variation of loss of balance and sparsity. These results were obtained from averaging over 200 independent random realizations of the ensembles.

Fig. 3 shows the root mean squared error of the first and second level intercept and slope parameters comparing the ground truth and the model estimations.

In general the change parameter estimates obtained from EM were found to be highly accurate, supporting the validity of the proposed method for different designs. As expected for a hierarchical model, the second level (group) parameter estimates were generally closer to the ground truth than first level (individual) change parameters. In our simulations, higher noise levels (or first level errors) primarily impaired first level parameter estimation accuracy.

To a minor extent, the first level noise also significantly affected the second level slope estimates, especially in sparse balanced designs. Similarly, larger individual differences (or second level errors) were found to increase estimation errors of the second level. Interestingly, larger individual differences also resulted in increased first level parameter errors, especially for less balanced designs.

We further found that having fewer follow up scans (or higher sparsity) in longitudinal designs broadly compromises individual and group level parameter estimates. Sparsity particularly affected all first level parameters in balanced and less balanced designs and the second level slope estimates; especially in balanced designs with more observational noise. In contrast, using more or less balanced designs had differential effects on estimation accuracy. Trajectory intercept errors were increased by more balanced designs, while slope estimates seemed at least in part to be improved.

**Fig. 4:**
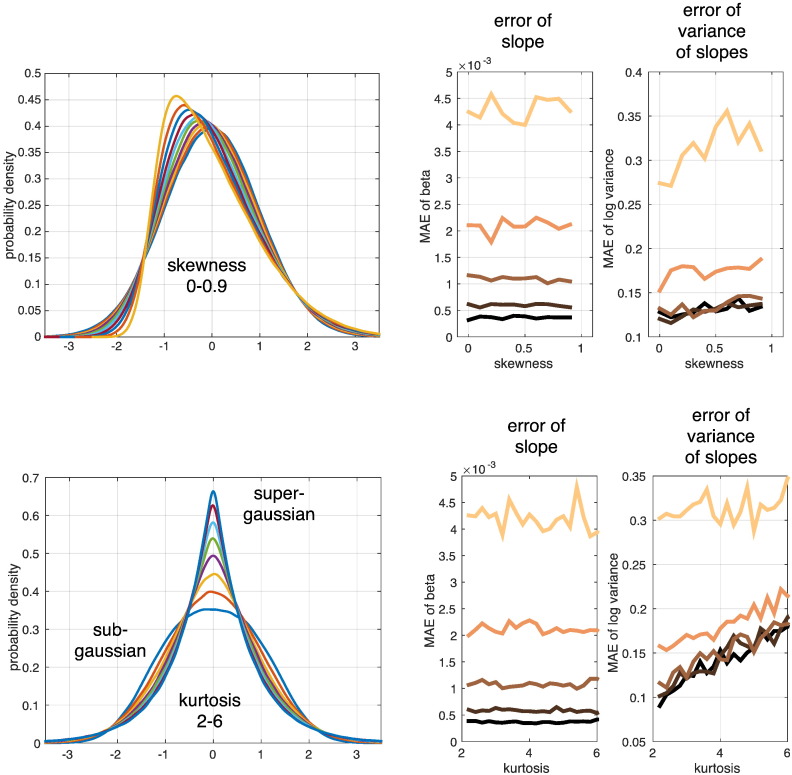
Effects of non-gaussianity for second level slope parameter and hyperparameter estimation accuracy. Generalized normal distributions (type I and II) were used for generation of trajectory data with non-Gaussian first and second level errors. We simulated ensembles of 64 subjects with 5 annual scans per person. These were sampled under balanced/unbalanced designs and linear Bayesian mixed-effects model inversion was performed. Skewness (top row) and kurtosis (bottom row) were independently manipulated from mean and variance. Estimated slope parameter and hyperparameter were compared to ground truth values computing the mean absolute error (MAE) over 200 independent realizations. Darker to brighter shading of MAE in plots depicts increasing first level errors std of 0.01, 0.02, 0.04, 0.08, and 0.16 respectively.

As our model is based on assumptions about Gaussian distributions, the model inversion and inference might be affected by any violation of this assumption. A second set of simulations was conducted to test the validity of our model inversion in the presence of non-Gaussian error distributions (Fig. 4). We explicitly manipulated skewness and kurtosis of the first and second level errors and assessed the stability and accuracy of group trajectory rate of change (slope) and the corresponding variability hyperparameter, i.e. *λ*_2_. Interestingly, we observed that rates of change in terms of group slope parameters were highly accurately reconstructed over a wide range of non-Gaussian distributions. Therefore, the main model parameter seemed to be almost completely unbiased. Furthermore, our results show a slightly biased estimation of the hyperparameters under higher values of skewness and especially large values of kurtosis, i.e. peaked or super-Gaussian distributions. However, in our experience, strongly super-Gaussian data is rather unlikely in deformation based morphometric features, while more often slightly skewed data due to modulations from jacobian determinants is observed. Additionally, given the empirical results, the posterior uncertainty was more strongly affected by the total variance differences of first and second level errors than by presence of significant higher central moments.

**Fig. 5:**
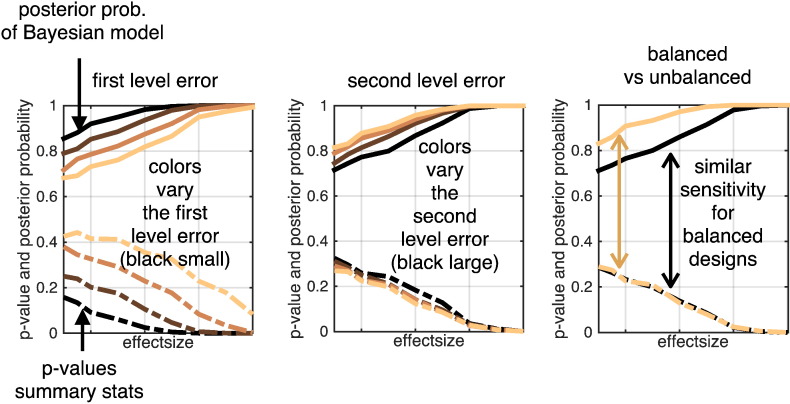
Comparison of Bayesian mixed-effects model with summary statistics for detection of changes on the group level, i.e. finding a negative slope for different ground truth effect sizes. Posterior probabilities (upper part) and p-values from summary statistics (lower part) are shown under variations of first (left) and second (middle) level error variances and design types (right). Summary statistics here means using independent linear models for every subject and calculating p-values from a one-sample *t*-test of obtained slope parameters. Realizations of ensembles of 64 subjects with 5 annual scans per person. These were sampled under balanced/unbalanced designs and subsequently modeled. Balanced here means that every subject has the same average age at measurements while unbalanced means a uniform distribution of each subject's average age across the whole study interval [20, 80]. All probabilities are shown as a function of (from left to right increasing) ground truth effect size, i.e. increasing steepness of decline. Results are obtained from averaging across 200 realization of ensembles for each parameter configuration. Color shading indicates the manipulation of the variable of interest, i.e. error sizes (left and middle) and balanced design property (right). Here, p-values and posterior probabilities show similar dependence on effect sizes in balanced designs (see black curves right plot). Posterior probabilities show a gain of sensitivity when designs become unbalanced (see ochre curves in right plot) while summary statistics perform similarly for both designs. Probabilities in left and middle plot are averages across multiple design types.

Finally, another possibility for evaluation and validation of our approach was used. We compared the linear Bayesian mixed-effects model to a simple summary statistic approach. The latter is generally valid if the design is balanced across subjects. That means that in this case the summary statistic approach should perform optimally, so we tested if our approach provides comparable results in this ideal scenario. As illustrated in Fig. 5, this idea was confirmed using a simulation framework with balanced and not age-balanced designs additionally varying the error variances. Our approach performed similarly to summary statistics for balanced designs over a wide variety of first and second level error variances. We also observed that Bayesian mixed-effects models appeared more powerful than summary statistics when the latter is expected to be sub-optimal, i.e. in unbalanced designs. A similar result was obtained comparing balanced designs and varying timing of observations on the within-subject level (not shown).

### Validation using real MRI data

#### Sample

In a second validation analysis, we provide a provisional assessment in terms of predictive validity by seeing if we could detect group differences (that we assume to be present). In this instance, we analyzed empirical data: The Bayesian mixed-effects models were applied and validated with a large longitudinal sample of healthy and pathological aging from the Alzheimer's Disease Neuroimaging Initiative (ADNI, http://www.adni-info.org)^3^ (see also Mueller et al., 2005).

We analyzed a subsample of the ADNI1 stage of the study, focusing on T1-weighted images acquired on 1.5 T scanners. After downloading and preprocessing 2397 scans of 474 participants, we excluded 39 subjects with 127 scans (due to substantial artifacts appearing in quality checks and errors during preprocessing). Apart from image sequence and preprocessing parameters (see also image preprocessing section), we did not apply any additional inclusion criteria.

The analyzed sample contained 2146 scans of 435 subjects, 181/254 female/male, ages 56.5–91.1, mean 76.4, std 6.7 years). The sample contains 10, 16, 31, 126, 113, 94, 43 and 2 subjects with ages 56–60, 60–65, 65–70, 70–75, 75–80, 80–85, 85–90 and 90–92 years respectively.

According to ADNI diagnostic criteria, the sample contained 688 scans of 140 healthy elderly subjects (further denoted as NO), 552 scans of 108 subjects with stable diagnosis of MCI during the whole ADNI study (denoted as sMCI), 530 scans of 92 subjects converting from original MCI diagnosis at baseline to AD during the ADNI study (pMCI), and 376 scans of 95 patients of patients diagnosed with AD.

The sample is less balanced with respect to age and the number of MRI acquisitions per subjects varies from 1 to 9 with 4.93 scans per subject on average. There were 34, 131, 122, 119 and 28 subjects having ≤ 3, 4, 5, 6 and ≥ 7 scans respectively. Most MRI acquisitions were performed at baseline or 6, 12, 18, 24, 36, 48, and 60 months of the within subject study time. The sample maps within subject healthy and pathological aging from 17, 32, 126, 218, 40 and 2 elderly subjects over 0–1, 1–2, 2–3, 3–4, 4–5 and 5–6 years respectively. A more detailed description of the ADNI study design and sample selection procedures can be found at http://adni.loni.usc.edu/data-samples/.

#### Symmetric diffeomorphic registration and image preprocessing

ADNI provides preprocessed T1-weighted images that have undergone specific correction steps to reduce scanner induced biases. To reduce these influences and minimize effects due to heterogeneity of protocols, all included images were chosen to match the MPRAGE with Gradwarp, B1 correction and N3 specification (see http://adni.loni.usc.edu/methods/mri-analysis/mri-pre-processing/). For further details about the applied ADNI MRI protocols please see http://adni.loni.usc.edu/methods/documents/mri-protocols/.

All further preprocessing steps were performed in SPM12b r6080 (Wellcome Trust Centre for Neuroimaging, London, UK, http://www.fil.ion.ucl.ac.uk/spm). Because longitudinal MR-based morphometry is particularly prone to artifacts due to scanner inhomogeneities, registration inconsistency, and subtle age-related deformations of the brains, it requires sophisticated preprocessing pipelines in order to detect the changes of interest and achieve unbiased results (Ashburner and Ridgway, 2013; Reuter and Fischl, 2011).

Thus, at first we applied the symmetric diffeomorphic registration for longitudinal MRI (Ashburner and Ridgway, 2013). In particular, this rests on a intra-subject modeling framework that combines non-linear diffeomorphic and rigid-body registration and further corrects for intensity inhomogeneity artifacts. The optimization is realized in a single generative model and provides internally consistent estimates of within-subject brain deformations during the study period. The registration model creates an average T1-image for each subject and the corresponding deformation fields for every individual scan.

Second, we applied SPM12b's unified segmentation to each subject's average T1-image, which assumes every voxel to be drawn from an unknown mixture of six distinct tissue classes: gray matter (GM), white matter (WM), and cerebrospinal fluid (CSF), bone, other tissue and air (see also Ashburner and Friston, 2005).

Third, all voxels within-subject average tissue maps were multiplied by the Jacobian determinants from the above longitudinal registration. Note, that this within-subject modulation is expected to encode all local individual volume changes during the study period.

Fourthly, nonlinear template generation and image registration was performed on the individual average GM and WM tissue maps using a geodesic shooting procedure (Ashburner and Friston, 2011). This defined the template space for all subsequent mixed-modeling steps.

Fifthly, the within-subject modulated (native space) segment images were subsequently deformed to this study template space. Note that only within- but no between-subject modulation was applied. We further quality checked the ensuing images manually and using covariance-based inhomogeneity measures as implemented in the VBM8 toolbox for SPM (http://www.neuro.uni-jena.de/vbm/).

Finally, images were smoothed using Gaussian kernels of 4 mm full width at half maximum. Subsequent modeling and analysis was performed for all tissue classes within corresponding binary masks. The masks were defined by a voxelwise sample mean of GM, WM and CSF tissue maps exceeding an absolute threshold of 0.1, 0.4, and 0.2 respectively.

All mixed-effects modeling steps were performed on 1.5 mm resolution images of ADNI subsamples using the above steps. The resulting images are assumed to reflect age-related effects, as well as healthy and pathological individual variability in terms of fine-grained maps of local gray matter (GMV), white matter (WMV) and cerebrospinal fluid volume (CSFV) content.

#### Computation time

Mass-univariate EM for Bayesian mixed-effects model inversion is computationally expensive. Single voxel computation time was found to depend on number of subjects, scans, groups, polynomial model degree and number of covariates. Subgroup models using linear trajectories (*N* = 60, *M* = 300, *G* = 1, *D* = 1, *R* = 1) took 4 h for local estimation in whole brain gray matter regions (0.05 s per voxel) on a desktop machine (6.5 CentOS Linux, Intel Xeon CPU, 3.20GHz, 12GB, Matlab R2013b). Using large sample data like ADNI with many hyperparameters, a single voxel inversion can take up to 30 s. However, mass-univariate estimation lends itself nicely to parallel computation. Using cluster computing facilities most model estimations were achieved within 1–3 days.

#### Normal aging and comparison of clinical group trajectories

**Fig. 6:**
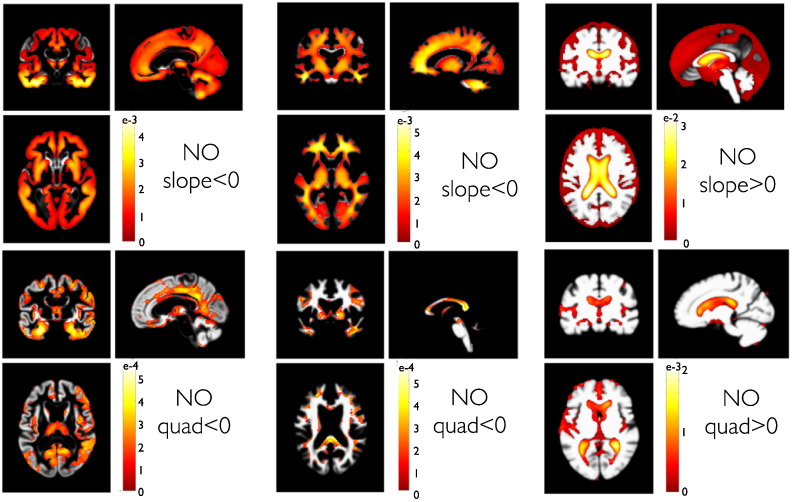
Group trajectories in 140 normal aging subjects (denoted as NO). PPMs are shown for the slopes and quadratic components in a second order model with *D* = 2. The PPM enables regionally specific inferences about parameter contrasts **c***^T^**θ*** and are shown after thresholding: showing only voxels for which the posterior probability *p*(*c*^*T*^***θ*** > 0|*y*) exceeds the probability 0.95 (with contrast vector c defining the effect of interest). For this particular comparison, the contrasts c contained an entry of one (or minus one) for the corresponding linear (top row) and quadratic (bottom row) second level normal aging group parameters and zero elsewhere. That means slope (and quadratic) < 0 denotes tests for linear (quadratic) components being smaller than zero. Color bar scaling denotes parameter contrast values **c***^T^**θ***, i.e. the slope or quadratic coefficients. White or gray colored regions have posterior probabilities < 0.95. The sign of the contrast is adapted to detect either decline in GM or growth in CSF volumes respectively. Columns depict PPMs of GM, WM and CSF tissue segments respectively.

First, we characterized trajectories in normal aging subjects. Fig. 6 shows PPMs of linear (i.e. slope) coefficients of the ensemble average trajectory in our normal aging group. In particular the PPMs indicated widespread decline of local volumes in GM and WM regions and substantial growth of CSF volume in the ventricles and sulcal regions. Using this sensitive longitudinal design, almost all regions were found to be affected by aging. Although the presented framework exploits linear mixed-effects models, one can explore nonlinear age-related effects by inclusion of quadratic terms and model. Assuming a quadratic model for every subject, we observe accelerated volume loss within many regions from all lobes. Most prominent accelerations were found in temporal GM and even more evident in the expansion of the lateral ventricle.

**Fig. 7:**
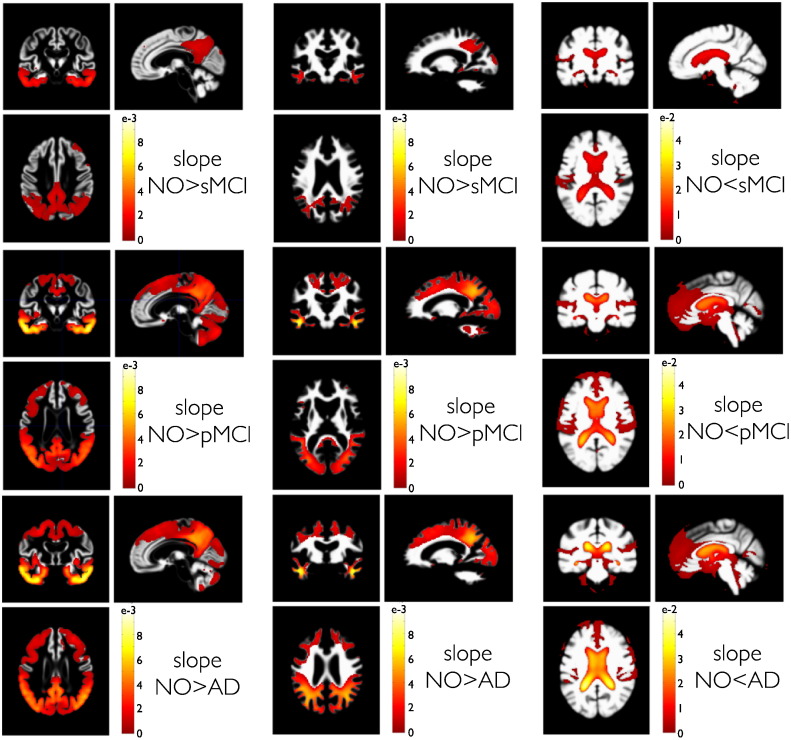
PPMs of clinical group trajectories compared to normal aging. PPMs are shown for differences of trajectory slopes in groups of 108 sMCI (top row), 92 pMCI (middle row) and 95 AD (bottom row) subjects compared to slopes in the NO group with 140 subjects. As with directed comparisons using one-sided t-tests in GLM, here we only depict the contrast for steeper slopes in the clinical groups. This contrast addresses the hypothesis that AD and MCI pathology produces faster volume loss for GM and WM volumes and faster volume increase in CSF volumes compared to normal aging. Columns depict PPMs of GM, WM and CSF tissue maps respectively. Color bars denote parameter contrast values **c***^T^**θ***, i.e. slope in NO minus slope in sMCI, pMCI and AD respectively. Because CSF shows growth, the sign of the contrast was reversed.

To further validate our model, we next compared local structural trajectories in clinical groups of the ADNI sample. Fig. 7 shows the PPMs of slope comparisons of the sMCI, pMCI and AD groups against the NO group. The comparisons of clinical and normal aging groups clearly indicate a region specific, temporo-parietal pattern of increased rates of atrophy in GM and WM volumes. This pattern is complemented by an increased rate of ventricle expansion in the disease groups. Groups that develop a full AD pathology (pMCI and AD) also show more negative rates of atrophy in frontal, occipital and cerebellar regions. Additionally to the more widespread spatial extent of the pathology in pMCI and AD compared to sMCI groups, the average rate of volume loss in terms of slope differences indicates a faster decline of regional temporo-parietal volume.

**Fig. 8:**
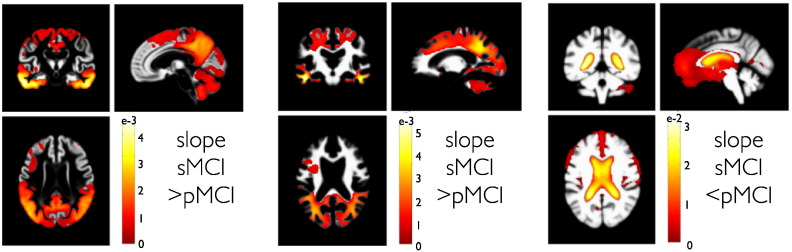
PPMs of stable MCI compared to progressive MCI group trajectories. PPMs are shown for differences of trajectory slopes in a group of 108 sMCI compared to 92 pMCI subjects. Here, we focus on the contrast for steeper slopes in pMCI compared to sMCI. Columns depict PPMs of gray matter, white matter and CSF tissue maps respectively. Color bars denote parameter contrast values **c***^T^**θ***, i.e. slope in sMCI minus slope in pMCI. Because CSF shows growth, the sign of the contrast was reversed.

**Fig. 9:**
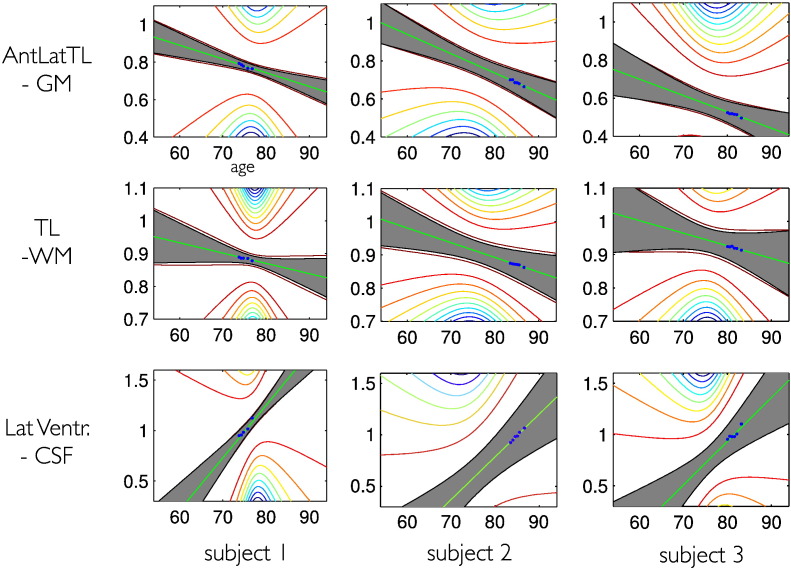
Individual structural trajectories using a linear model. Three random subjects (1 NO, 1 sMCI, 1 pMCI) were chosen and we demonstrate local trajectories in three example voxels from the anterior lateral temporal lobe GM (upper row), temporal lobe WM (middle row), and lateral ventricle (bottom row). The observed data is shown in blue, the individual predicted trajectory *g*(*t*, ***θ***_*i*_^(1)^) is shown in green including the ± 2 standard deviation of its posterior uncertainty (gray area) and the contour plot of the uncertainty pdf outside the ± 2 std area. The uncertainty is mainly driven by the parametrization around the center of mass of age *r_t_* in the whole group.

This conversion effect can be seen by evaluation of slope differences in pMCI and sMCI groups (Fig. 8). According to our sample, the conversion from MCI to AD at some point during the study also seems to be reflected in differential rates of local brain volume changes. Due to limitations of space, we restrict our presentation of comparisons to second level slope parameters. It is worth mentioning that the model supports similar comparisons for the trajectory intercepts, which mainly reflect existing differences before the study, as opposed to ongoing changes of brain structure during the study. Three examples of individual structural trajectories are shown in Fig. (9).

**Fig. 10:**
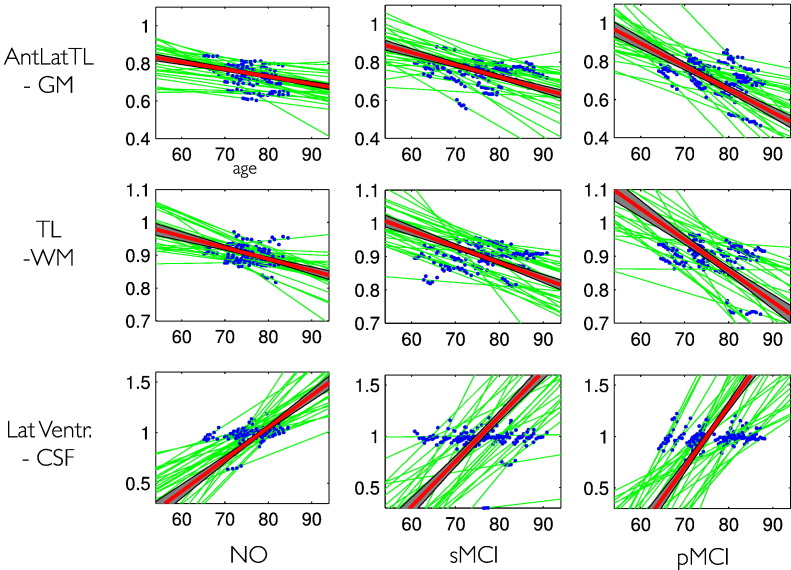
Individual and group level structural trajectories using a linear model. Three single voxels (same as in 7) were chosen to demonstrate our local trajectory model: anterior lateral temporal lobe GM (upper row), temporal lobe WM (middle row), and lateral ventricle (bottom row). The observed data is shown in blue, the individual predicted trajectories *g*(*t*, ***θ***_*i*_^(1)^) are shown in green. The group average trajectories are shown in red attached with the ± 2 standard deviation of its posterior uncertainty (gray area). To improve visualization, only 30 individual trajectories (without uncertainty) are shown.

Examples of subject level and group level trajectories in NO, sMCI, and pMCI groups are displayed in Fig. 10. As expected for a hierarchical model, the posterior trajectory precision (or inverse variance) is found to be much smaller for the group level compared to the individual level. Ensemble trajectory estimates in groups are more precise and inference therefore more sensitive for detecting developmental differences.

#### Analysis of individual differences of trajectories

In contrast to typical cross-sectional MRI studies of brain development and aging, individual trajectory models, based on repeated measures MRI, also afford analysis of within-subject change variability. A strength of our approach is that we can explore effects of risk or protective factors on ongoing structural decline. This could involve lifestyle parameters, genetic profiles, cognitive test scores etc. or any stable between-subject variable of interest.

**Fig. 11:**
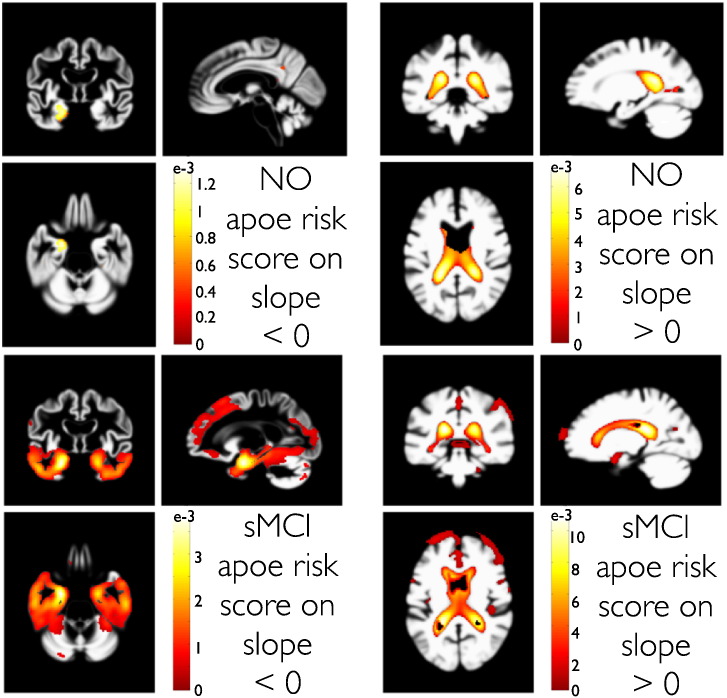
Parametric analysis of trajectory slope variability using an ApoE4 risk score in NO (top row) and sMCI (bottom row) subjects. The applied ApoE4 risk score counts the number of ApoE4 alleles, 0, 1, or 2 respectively. PPMs with contrasts for a steeper local GMV decline (left column) and CSFV growth (right column) with increased risk are shown. Color bars denote parameter contrast values *C**θ***, with *C* containing a (minus) one for the ApoE4 regressor and zero elsewhere.

To demonstrate the potential of this method, we focused on explaining variability of local rates of atrophy based on the E4 allele of the Apolipoprotein gene (further denoted as ApoE4), an established risk factor for increased lifetime prevalence of AD. We define this score as the number of ApoE4 alleles of an individual, which can have either zero, one or two copies. This risk score was entered as a predictor **Z** (in Eq. (6)) for slope variability. Fig. 11 shows the PPMs for voxels showing steeper decline of GMV (or growth of CSFV) with higher ApoE4 risk in the group of NO and sMCI.

We observed localized effects indicating faster volume loss in anterior medial temporal lobe regions and lateral ventricle growth in normal subjects with higher ApoE4 risk scores. More widespread effects were found in temporo-parieto-frontal GM regions of stable MCI subjects. In addition to the above group differences of change, these results demonstrate the sensitivity of our method for analysis of additional within-group heterogeneity of change.

#### Comparing models of different degrees

Here we demonstrate examples for evidence-based model comparison within our generative trajectory modeling framework. There are many questions in the context of longitudinal MRI studies that can be elegantly framed in terms of model comparisons.

Firstly, one might aim at inference about different parametrizations, particularly the choice of a certain polynomial model degree of random and fixed effects of the trajectory models, i.e. the choice of [*D*, *D*_*f*_]. This is crucial in light of evidence for nonlinearities in brain maturation (Shaw et al., 2008), accelerated gray matter loss in healthy aging (Fjell et al., 2013) and other nonlinearities in clinical groups (Leung et al., 2013).

**Fig. 12:**
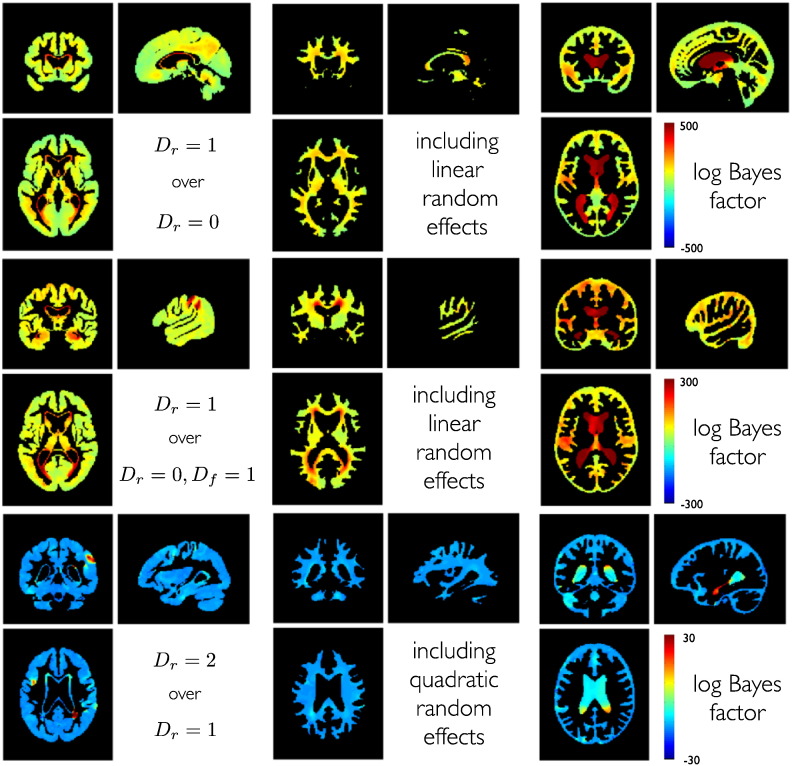
Bayesian model comparison about polynomial degree within the normal aging group with N = 140. Top row shows log Bayes factors comparing a model with random intercepts and random slopes to a model with only random intercepts (*D* = 1 vs. *D* = 0). Middle row shows the comparison of a random intercept and random slope model to a random intercept model with fixed effects slope (*D* = 1 vs. *D* = 0, *D_f_* = 1). Bottom row shows log Bayes factors comparing a full second order random effects model compared to a linear random effects model with random slopes and intercepts (*D* = 2 vs. *D* = 2). Columns show model comparisons separately for gray matter, white matter and CSF. Higher mixed-effect degrees were estimated but are not shown because of lower model evidence and limitations of space.

Secondly, one might also be interested in comparing generative models using different sets of covariates, e.g. by including informative predictors for individual differences of change. Fig. 12 gives an example of log Bayes factors for linear and second order models obtained from independent EM optimization for each model. Bayes factors in our normal aging group clearly favor a linear random effects model over alternative models in most gray matter regions. Introducing age as a fixed effect increased model evidence. Model evidence was further improved by allowing for random slope variability in most gray matter regions, especially in medial temporal lobe regions. According to the same comparison, individual differences among structural brain changes are most pronounced in the lateral ventricle regions.

Interestingly, parts of the ventricles exhibited further increased model evidence by additional inclusion of random quadratic growth effects. This was found to be emphasized for the lateral ventricle which in parts borders on the hippocampus. We further evaluated models with all combinations of fixed and random effects up to second order.

The overall winning model in most gray matter includes random effects for intercepts and slopes. Exceptions were found in right temporo-parietal and postcentral gray matter regions, and in left inferior frontal gyrus. Here and within parts of the lateral ventricle a quadratic random effects model was better able to capture individual differences of change in normal aging.

#### Permutation testing for empirical false positive rate

**Fig. 13:**
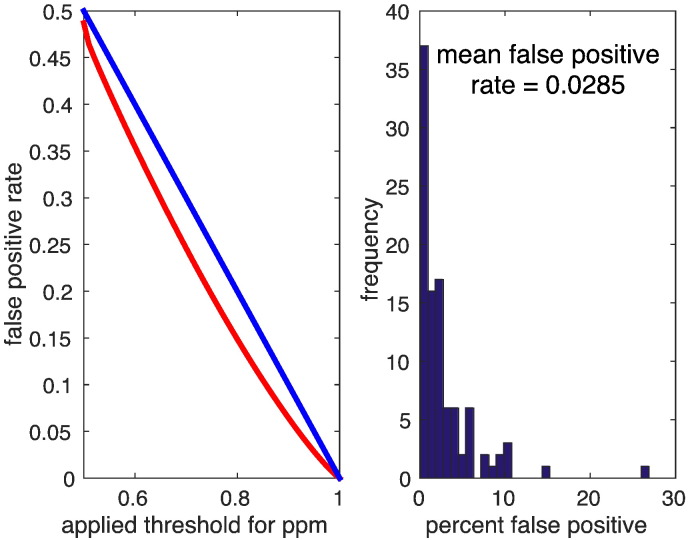
Empirical false positive rate (red) obtained from permutation testing. We used 100 random permutations of a subset of 60 normal subjects with the original design and subsequently inverting the model including 300 000 gray matter voxels. Posterior probabilities were thresholded using various thresholds (e.g. 0.95) and false positive rate was estimated as the number of above threshold voxels per volume averaged over the all permutations. The histogram of obtained false positive rate is shown right.

Finally, to ensure our voxelwise estimation scheme does not produce spurious or misleading conclusions we repeated a similar analysis under random permutations. We focussed on a subsample of 60 normal subjects with 300 MRI scans. Similar effect maps as shown for the whole group of normals were obtained. Then the data was randomly permuted 100 times and we reran the Bayesian model inversion outlined above. Posterior probability maps were calculated exactly as outlined in the full ADNI model. We hoped to see that the number of voxels in the ensuing PPMs (thresholded at 95%) was 5% of the search volume or less. The mean false positive rate was found to be 2.85%. The distribution of % suprathreshold voxels over 100 presentations (with replacement) is shown in Fig. 13 (right). More generally, no indication for increased false positive rates was found for other probability thresholds as well (see Fig. 13 left).

## Discussion

We have described, validated and applied a powerful framework for analysis of brain morphometry in longitudinal MRI data using Bayesian inference. The emphasis is on the analysis of individual differences of brain changes in one or more samples and subsequent inference about the contribution of subject specific covariates such as cognitive abilities, behavior, psychopathology, health, and lifestyle factors.

In particular, the approach exploits algorithms for within- (Ashburner and Ridgway, 2013) and between- (Ashburner and Friston, 2011) subject diffeomorphic registration in order to generate non-linearly registered tissue volume images of subjects and scans using Jacobian determinants of deformations. The resulting (modulated) tissue segment maps are subjected to mass-univariate generative mixed-effects modeling.

EM is used for Bayesian inversion of the generative model by estimating variance components and empirical Gaussian priors on individual differences of change. The model is hierarchical and provides estimates of local individual change trajectories over the whole study period, even for variable numbers of scans per subject or for less balanced designs.

Our approach is similar to recently proposed iterative schemes for surface-based cortical thickness analysis in longitudinal MRI data (Bernal-Rusiel et al., 2012, 2013) and fMRI group analysis (Chen et al., 2013). We also briefly compared our EM algorithm to the openly available mass-univariate mixed-effects algorithms from Freesurfer (https://surfer.nmr.mgh.harvard.edu/fswiki/LinearMixedEffectsModels) (Bernal-Rusiel et al., 2012) (not shown in results) using synthetic longitudinal data (from the section on Validation using simulated structural trajectories ) with linear models and balanced data with Gaussian errors. We found convergence to the same group trajectory parameter estimates suggesting the validity of the applied iterative mixed-model schemes. However, a detailed evaluation of multiple approaches in multiple settings (a) including non-Gaussian error distributions (b) with both balanced and unbalanced designs (c) with linear and non-linear ground truth trajectories, is left for future work.

In contrast to the above methods, our model focuses on Bayesian inference on fixed- and random-effect parameters for individual and group trajectories, as well as Bayesian model selection. Our focus on Bayesian inference and random effects models overcomes some limitations of classical inference (for discussion see Friston et al., 2002a) and the proposed evidence based comparison of models allows one to navigate a rich model space.

Other Bayesian approaches for longitudinal MRI have been proposed with emphasis on either classification, evaluation of treatment effects, and dynamical networks (Elliott et al., 2010; Schmid et al., 2009; Chen et al., 2012). Our approach complements these methods by providing single subject trajectories and a model of their heterogeneity in aging samples. Notably, our iterative EM based random effects estimation also substantially differs from non-iterative marginal modeling using generalized estimating equations (for introduction see eg. Fitzmaurice et al., 2008) with recent application to longitudinal data using sandwich estimators (Guillaume et al., 2014) and adaptive multiscale methods (Skup et al., 2012).

We extensively validated our method using ground truth comparison with simulated longitudinal data. The model consistently reproduced veridical estimates across study designs with different characteristics. A design with fewer scans per subject was found to substantially reduce parameter accuracy, especially for the rates of change (or slopes). This result favors less sparse designs for efficient analysis of individual differences of change. Less balanced designs were also found to increase deviations from ground truth with some exceptions, especially for second level slope estimates and higher noise levels.

Notably, by construction, the design variability is part of the likelihood model and these effects are fully accounted for in the posterior parameter uncertainty (or credible intervals). Thus, using PPMs is expected to provide valid inference about individual, group and covariate parameters across a wide variety of study designs. Moreover, using non-Gaussian distributions, we have revealed some evidence for the robustness of the method under potential violations of the normality assumption. Mean parameter estimates were found to be unaffected from non-Gaussianity, hyperparameters were rather mildly affected by skewness and more biased by very large values of kurtosis. Comparison to summary statistics showed that posterior probabilities perform similarly in balanced designs and are likely to improve inference in typical unbalanced observational designs.

We further validated our approach using real MRI data from a large subsample of the available ADNI dataset. The spatio-temporal pattern of structural trajectories in subsamples for Normal Aging, stable MCI, progressive MCI and AD was found to be consistent with existing neuroimaging evidence (Driscoll et al., 2009; Misra et al., 2009; Anderson et al., 2012; Barnes et al., 2008; Vemuri et al., 2010b). Applying linear models of trajectories, PPMs of clinical groups indicated substantially increased rates of local brain atrophy and ventricle growth. The spatial pattern clearly emphasizes temporo-parietal regions in stable MCIs compared to normal aging, while higher rates of atrophy in pMCI and AD were also found in frontal gray matter regions. The sensitivity of this longitudinal mixed-modeling method was further demonstrated by observing differential rates of atrophy in progressive MCI compared to stable MCI. In line with recent evidence in healthy aging (Fjell et al., 2014), we also found additional accelerated decline (i.e. reverse U-shaped trajectories) of cortical and subcortical gray matter regions and accelerated growth (i.e. U-shaped trajectories) of lateral ventricle using quadratic models. As suggested by the study of Holland et al. (2012), different patterns of change of rates of atrophy might apply to pathological compared to healthy aging groups. We will focus on the volume dynamics during disease progression in a separate paper.

Using ADNI data we also aimed to explore the strength of mixed-effects models to identify the effects of covariates of interest. For that particular purpose, we chose to analyze the effects of a genetic risk score based on the number of ApoE4 alleles, i.e. 0, 1, or 2, a well known and established risk factor for development of AD and corresponding signs of atrophy in MRI (Vemuri et al., 2010a; Risacher et al., 2010; Morgen et al., 2013; Taylor et al., 2014; Tosun et al., 2010; Moffat et al., 2000; Hostage et al., 2014). Although one could have alternatively used group comparisons based on number of ApoE4 alleles, we preferred to include this risk score as an example for an additional predictor of within group variability around the mean changes in normal aging and stable MCI groups.

The PPMs of ApoE4 risk's second level contrast indicated effects on variability of ventricle growth in normal aging and widespread effects on gray matter rate of atrophy in stable MCI. This emphasizes the risk score as an important contributing factor to local structural aging. Similarly, this technique could be used for parametric analysis of other risk scores or continuous behavioral variables thought to be involved in development and aging.

Candidate hypotheses about brain development and aging can be framed in terms of specific trajectory models. These hypotheses might involve (A) the inclusion of certain degrees of fixed or random effects of time, nonlinearities etc. and (B) explicitly modeling brain–behavior relationships by inclusion of behavioral covariates. Scientists can then use Bayesian inference to update their beliefs about the respective hypotheses, in light of new (neuroimaging) data.

Bayesian model selection has been introduced to identify the most likely of a set of hypotheses e.g. using log model evidence ratios or Bayes factors (Kass and Raftery, 1995). Evidence comparisons of nested models are analogous to the F-tests commonly used in SPM (Friston et al., 1995). However, a major advantage of applying Bayesian instead of frequentist inference to trajectory models is that evidence based comparison extends to non-nested models. This is useful because different combinations of random and fixed effects or covariates are not necessarily nested. For instance first order random effects models cannot be reduced to a zero order random effects model with first order fixed effects by setting some variables to zero.

Voxelwise model evidence maps were previously introduced for efficient group level inference in fMRI using random effects (Rosa et al., 2010). Our models extend this idea to mixed-effects models for longitudinal MRI. Using a normal aging sample, we here demonstrate that Bayesian model selection can be also used for particular choices of combinations of random and fixed effects in normal aging-related structural changes. We explored a model space with all combinations of fixed and random effects up to order three. Pairwise comparisons of models were illustrated using Bayes factor maps.

The model with the most evidence was found to vary over regions and tissue classes. For most gray matter regions a random intercept and slope model was found to be most likely, with exceptions of a left prefrontal and a postcentral region, and regions adjacent to the ventricles. These were found to show second order random effects with individual differences of accelerations.

The second order random effects model was also more likely for the lateral ventricle adjacent to the hippocampus and its posterior parts. Although we only found accelerated lateral ventricle volume increases, this is in line with recent observations of late accelerated aging in hippocampal gray matter in normal aging (Ziegler et al., 2012b; Fjell et al., 2014). Disregarding potential segmentation difficulties of hippocampal gray matter, one also might expect that the spatial regularization of the within-subject deformations is slightly biased towards the adjacent ventricle growth. This might have reduced the sensitivity for detection of second order individual decline differences in hippocampal regions.

At the same time, our results extend existing fixed effects findings. Similar to a recent study using ROIs from manual volumetry (Raz et al., 2010), mixed-effects models go beyond testing for (nonlinear) fixed effects of aging because they explicitly model heterogeneity of structural changes.

In contrast to findings of Raz et al. (2010), where some regions did exhibit age-related change, but without any sign of individual differences, here Bayesian model selection showed the highest model evidence for linear or even quadratic random effects. In fact, in our voxelwise whole brain search we did not observe any brain region in all three analyzed tissue classes that exhibited most evidence for a model with random intercepts and linear fixed effects, i.e. showing uniform aging across subjects. These deviations of semi-automated and manual longitudinal volumetry might be further addressed.

Our ADNI sample findings suggest substantial heterogeneity among local structural brain changes in normal aging subjects without (or prior to) signs of dementia. Similar questions might be addressed about the heterogeneity of trajectories in disease states and during treatment processes.

It is also worth mentioning that the optimal degree of random effects (from evidence based comparison) specifies the dimensionality of individual differences in aging brain structure. This degree determines the complexity of a sufficient individual model of change rather than only quantifying the smoothness of the temporal dynamic on the group level. This idea nicely connects to the multivariate perspective on cognitive ability differences (see e.g. Ziegler et al., 2013).

Future studies might focus on Bayesian model selection in larger random effects model spaces using additional sets of genetic, physiological, and behavioral predictors. After sufficiently capturing the complexity of individual differences of aging-related brain changes as random effects (or hidden variables), one might aim to explain latent variables based on other observations, such as behavior, genes, and other MRI modalities.

Here we applied uninformative priors in the presented results. However, the proposed framework enables flexible specification of prior structures at the top-level of parameters, which can be used to implement prior knowledge about the process of interest, e.g. in terms of expected growth or decline rates in development or aging. The framework (and the corresponding implementation in SPM) will provide the choice of top-level priors being either uninformative (i.e. flat) or informative. Uninformative priors can be used for exploratory research similar to other standard mixed-effects models, while otherwise, informative priors can be chosen to be either fixed (for fully Bayesian inference) or estimated from the data using empirical Bayes. In particular, further extensions aim to include examples of empirical priors, e.g. global shrinkage or atlas-based regional shrinkage priors which regularize all voxelwise trajectory estimates after estimating a top level prior on the whole brain or regional ROI level respectively. The use of empirical priors in context of neuroimaging data was recently motivated by machine learning applications showing the potential for probabilistic single case inference given the ‘prior-knowledge’ of a large MRI database (Ziegler et al., 2014). Although we did not observe any evidence for increased rates of false positives during permutation testing, it is worth mentioning that empirical priors have also been discussed in the context of control of false discovery rate (FDR) (Schwartzman et al., 2009).

We finally like to mention some limitations and possible extensions of this work. Firstly, Bayesian model reduction has been recently proposed for efficient inference on general linear models and dynamical systems models of neuroimaging data (Friston and Penny, 2011; Penny and Ridgway, 2013). Using model reduction, posterior estimates and model evidences might be accurately approximated for large model spaces using only the optimized full model (instead of inverting every reduced model). Future studies might therefore work on efficient approximation techniques to improve the efficiency of Bayesian model comparison across wider spaces of mixed-effects models.

Secondly, our presented model applied group specific priors with independent estimation of multiple ensembles of trajectories. However, the hierarchical modeling framework naturally extends to higher levels. These could be extended to model individual differences of changes in multiple clinical groups of a joint population, the inclusion of multi-center scanner levels, and the variance across birth cohorts.

Thirdly, the mass-univariate Bayesian model inversion is computationally very expensive and does not account for spatial correlations among the voxels. As in recent work, the model might be extended to combine priors on heterogeneity and image space using full spatio-temporal-models or adaptive smoothing techniques (see e.g. Bernal-Rusiel et al., 2013; Skup et al., 2012).

Finally, recently developed techniques in quantitative imaging provide biologically relevant properties, e.g. about brain myelination and iron levels (Draganski et al., 2011; Callaghan et al., 2014; Lambert et al., 2013). Following quantitative biomarkers in healthy and pathological development might be expected to provide biologically meaningful models of developmental heterogeneity while reducing the potential influence of anatomical shape variability.

## Footnotes

2 Please note that the actual implementation uses $\Delta \lambda ={\bar{H}}^{-1}g$ with ${\bar{H}}_{\mathrm{kl}}=H_{\mathrm{kl}}-P_{h}$ and hyperpriors *P*_*h*_ = 1/32. The treatment of hyperparameters using a probabilistic perspective is motivated within the variational Bayes framework (Friston et al., 2007) and increases numerical stability of the optimization scheme.

3 Data used in the preparation of this article were obtained from the Alzheimer's Disease Neuroimaging Initiative (ADNI) database (adni.loni.usc.edu). The ADNI was launched in 2003 by the National Institute on Aging (NIA), the National Institute of Biomedical Imaging and Bioengineering (NIBIB), the Food and Drug Administration (FDA), private pharmaceutical companies and non-profit organizations, as a $60 million, 5-year public–private partnership. The primary goal of ADNI has been to test whether serial magnetic resonance imaging (MRI), positron emission tomography (PET), other biological markers, and clinical and neuropsychological assessment can be combined to measure the progression of mild cognitive impairment (MCI) and early Alzheimer's disease (AD). Determination of sensitive and specific markers of very early AD progression is intended to aid researchers and clinicians to develop new treatments and monitor their effectiveness, as well as lessen the time and cost of clinical trials. The Principal Investigator of this initiative is Michael W. Weiner, MD, VA Medical Center and University of California – San Francisco. ADNI is the result of efforts of many co-investigators from a broad range of academic institutions and private corporations, and subjects have been recruited from over 50 sites across the U.S. and Canada. The initial goal of ADNI was to recruit 800 subjects but ADNI has been followed by ADNI-GO and ADNI-2. To date these three protocols have recruited over 1500 adults, ages 55 to 90, to participate in the research, consisting of cognitively normal older individuals, people with early or late MCI and people with early AD. The follow up duration of each group is specified in the protocols for ADNI-1, ADNI-2 and ADNI-GO. Subjects originally recruited for ADNI-1 and ADNI-GO had the option to be followed in ADNI-2. For up-to-date information, see http://www.adni-info.org.
